# Increased Resting-State Functional Connectivity in Patients With Autoimmune Addison Disease

**DOI:** 10.1210/clinem/dgad592

**Published:** 2023-10-11

**Authors:** Annelies van’t Westeinde, Nelly Padilla, Sara Fletcher-Sandersjöö, Olle Kämpe, Sophie Bensing, Svetlana Lajic

**Affiliations:** Department of Women's and Children's Health, Karolinska Institutet, Pediatric Endocrinology Unit, Karolinska University Hospital, SE-171 76 Stockholm, Sweden; Department of Women's and Children's Health, Karolinska Institutet, Unit for Neonatology, Karolinska University Hospital, SE-171 76 Stockholm, Sweden; Department of Molecular Medicine and Surgery, Karolinska Institutet and Department of Endocrinology, Karolinska University Hospital, SE-171 76 Stockholm, Sweden; Department of Medicine (Solna), Center for Molecular Medicine, Karolinska Institutet, Sweden and Department of Endocrinology, Karolinska University Hospital, SE-171 76 Stockholm, Sweden; Department of Molecular Medicine and Surgery, Karolinska Institutet and Department of Endocrinology, Karolinska University Hospital, SE-171 76 Stockholm, Sweden; Department of Women's and Children's Health, Karolinska Institutet, Pediatric Endocrinology Unit, Karolinska University Hospital, SE-171 76 Stockholm, Sweden; Department of Clinical Sciences, Sahlgrenska Academy, University of Gothenburg, Pediatric Endocrinology Unit, Sahlgrenska University Hospital, SE-416 50 Gothenburg, Sweden

**Keywords:** Addison disease, cortisol, resting-state functional connectivity, default mode network, orbitofrontal

## Abstract

**Context:**

Individuals with autoimmune Addison disease (AAD) take replacement medication for the lack of adrenal-derived glucocorticoid (GC) and mineralocorticoid hormones from diagnosis. The brain is highly sensitive to these hormones, but the consequence of having AAD for brain health has not been widely addressed.

**Objective:**

The present study compared resting-state functional connectivity (rs-fc) of the brain between individuals with AAD and healthy controls.

**Methods:**

Fifty-seven patients with AAD (33 female) and 69 healthy controls (39 female), aged 19 to 43 years were scanned with 3-T magnetic resonance imaging (MRI).

**Results:**

Independent component and subsequent dual regression analyses revealed that individuals with AAD had stronger rs-fc compared to controls in 3 networks: the bilateral orbitofrontal cortex (OFC), the left medial visual and left posterior default mode network. A higher GC replacement dose was associated with stronger rs-fc in a small part of the left OFC in patients. We did not find any clear associations between rs-fc and executive functions or mental fatigue.

**Conclusion:**

Our results suggest that having AAD affects the baseline functional organization of the brain and that current treatment strategies of AAD may be one risk factor.

Autoimmune Addison disease (AAD) is a form of primary adrenal insufficiency in which the adrenal cortex is destroyed by an autoimmune attack. This results in chronic glucocorticoid (GC) and mineralocorticoid (MC) deficiency, which is life-threatening if not treated promptly (1). Patients start medication on diagnosis, consisting of life-long replacement therapy, usually in the form of oral immediate-release hydrocortisone (IR-HC) 2 to 3 times daily and 9-α-fludrocortisone, once daily (1). The adrenal cortex also produces androgens such as DHEA (dehydroepiandrosterone). In female patients, the loss of adrenal androgens entails at least a 50% loss of total testosterone levels (2). However, adrenal androgens are replaced in only a minority of female patients (3). Compliance with treatment is needed to prevent life-threatening adrenal crises, in which the patient experiences acute cortisol deficiency that may lead to circulatory shock and death. Replacement doses are currently advised to be the lowest dose possible on which the patient feels well to prevent negative side effects from cortisol overdosing (4). However, replicating hormonal secretion is difficult. Thus, patients on current replacement therapy lack both the natural ultradian and diurnal rhythm of cortisol, and theoretically have reduced flexibility of the hypothalamus-pituitary-adrenal axis as they cannot produce cortisol in response to internal or external stressors (5). Individuals with AAD therefore need to take higher doses of hydrocortisone in times of stress such as during febrile episodes and surgery. The IR-HC formulas further result in sharp peaks and troughs in GC levels. Oral modified-release hydrocortisone (MR-HC) formulas aim to produce a smoother release but further diminish the ultradian rhythmicity (6, 7). Due to the widespread effects of cortisol on the body, supraphysiological and infraphysiological cortisol levels may affect many physiological functions.

The brain widely expresses both GC and mineralocorticoid receptors, and it is well known that cortisol can affect both the structure and the functional activity and connectivity of the brain on a short-term and long-term basis (8–10). Cortisol disturbances lead to sleep problems (5, 11) and affect many cognitive and affective processes, including long-term memory, working memory, and emotion regulation, in particular in response to stressful situations, which may be underpinned by changes in brain structure and function (12–14). These topics have been scarcely addressed in the case of Addison disease, while insight into brain health could give clues for optimizing the treatment and help improve patient well-being and maintain optimal cognitive functioning in the long-term. Some studies in AAD have found problems with cognitive functioning in patients, but the effects seem mild and are not found consistently (15–19). However, patients do have sleep disturbances and fatigue (20) that might mediate problems with cognition (16), and they self-report mental fatigue and difficulties with executive functioning, especially women (19). Mental fatigue was the strongest predictor of experienced executive function problems in our cohort (19). No neuroscientific studies exist yet that have investigated potential brain function alterations underlying these issues. The only brain imaging study on AAD found around 4% smaller total brain volume (BV) in patients, with higher GC replacement dose being associated with smaller BV and smaller volume of a few regions, namely the left lingual gyrus, left rostral anterior cingulate cortex (ACC), and right supramarginal gyrus, but there were no differences in white matter microstructure (21). The changes were relatively small, in particular compared to patients with other cortisol-related diseases such as Cushing disease and congenital adrenal hyperplasia (CAH), who show more elaborate and profound alterations in both gray and white matter (22–25). However, changes in brain activity and functional connectivity may still arise in individuals with AAD, for example, as a compensation for or maladaptive result of the change in structure, or as an independent effect on neural network organization of the long-term hormonal imbalances and acute events such as adrenal crises.

Studies on long-term cortisol disturbances in CAH and Cushing disease have all reported changes in resting-state functional connectivity (rs-fc) in various networks (26–29). In Cushing disease, time since remission as well as blood cortisol levels are associated with strength of rs-fc (26–28). Van der Werff and colleagues (27) also assessed correlations with cognitive performance but found no significant associations, which they thought suggests that the alterations are part of some compensation mechanism. However, none of these studies addressed fatigue. In healthy people, fatigue has been found to be associated with increased activity and connectivity in the default mode network (DMN), both during and after performing a task (30), which might be part of a compensation mechanism associated with increased motivation. Hence, we may expect changes in rs-fc in individuals with AAD, in particular in relation to experienced mental fatigue.

Brain activity may further be differentially modulated based on sex, given the relatively disproportionate loss of androgens in female patients.

The present study sought to investigate rs-fc of the brain in a cohort of young individuals with AAD. In addition, we aimed to investigate the modulating effect of sex and the association between rs-fc and mental fatigue, executive function, and disease-related factors such as GC replacement dose and disease duration.

## Materials and Methods

### Participants

Participants were part of a larger study on primary adrenal insufficiency (CAH and AAD) (31). Here, we compare individuals with AAD with healthy controls from the Swedish population (31). Inclusion and exclusion criteria are described in (19, 21). Seven controls and 6 individuals with AAD did not participate in the mineralocorticoid receptor part of the study protocol. After rs and structural magnetic resonance imaging (MRI) scan preprocessing and quality control, the final cohort for this report consisted of n = 57 (33 female) individuals with AAD and n = 69 (39 female) controls, aged 19 to 43 years. Twenty-two patients had comorbid hypothyroidism. Forty-five patients were treated with IR-HC (2 or 3 daily doses), and 12 patients with MR-HC (Plenadren) once daily. Seven female participants received DHEA treatment. Fourteen patients had been diagnosed with AAD before age 18 years. All participants gave written informed consent to take part in the study. The study was approved by the regional ethical committee of Karolinska Institutet and by the Swedish Ethical Review Authority (DNR 99-153 [990517]; 030619; 2011/1764-32 [111201]; 140912; 2017/1658-32 [170831]; 2018/1037-32 [180518]; 2020-00564 [200414]).

### Procedures

Participants underwent MRI of the brain, performed neuropsychological tests, and filled out self-rating questionnaires (19). For the present study, we used a test of visuospatial working memory performance, namely the Span Board Test forward and backward (32), converted to scaled scores (population norm M = 10, SD = 3), verbal working memory (Wechsler Adult Intelligence Scale [WAIS-IV Digit Span] (33)), self-reported experienced difficulty with executive function in the past 2 weeks (Barkley Deficits in Executive Functioning Scale short form [BDEFS-SF] (34)), and self-reported mental and general fatigue during the past 2 days on the Multidimensional Fatigue Inventory (MFI) (35).

### Demographics Analyses

All statistical analyses were conducted using the open source R software, version 3.6.1 (36). Group differences were determined between patients and controls in proportion of male and female participants, education level (higher education defined as having completed at least 3 years of university studies) (chi-square tests), illegal drug use (Fisher exact test), age, and alcohol use (Wilcoxon test for nonparametric data); and within the AAD patient group, sex differences regarding hypothyroidism (chi-square tests), total hydrocortisone replacement dose (either IR-HC or MR-HC estimated as mg HC eq/m^2^/day (37)), number of adrenal crises, age of disease onset, and disease duration (Wilcoxon test for nonparametric data).

### Magnetic Resonance Imaging Data Acquisition and Analyses

MRI scans were acquired on a 3-T MR scanner (Discovery MR750, General Electric) with an 8-channel head coil during a 70-minute session. We acquired images with T1 and diffusion-weighted imaging, in addition to functional MRI (fMRI) during 2 working-memory tasks and 1 resting-state scan. For the present paper, we analyzed data from the 8-minute, eyes-closed rs functional scan (gradient echo sequence, echo planar imaging, repetition time [TR] = 2000 ms, echo time [TE] = 30 ms, voxel size 3.0 × 3.0 × 3.0 mm^3^, 41 slices, flip angle = 70°), in addition to using anatomical T1-weighted images (T1-weighted BRAVO sequence, TR = 7.9 ms, TE = 3.1 ms, 176 slices, voxel size: 1.0 × 1.0 × 1.0 mm^3^). During the rs scan participants were instructed to keep their eyes closed but not fall asleep, and not think of anything in particular, but instead just let their thoughts wander.

#### Preprocessing resting-state functional images

rs functional MRI data were preprocessed using FMRIB’s Software Libraries version 5.0.11 (FMRIB Laboratory, University of Oxford) (38). Before processing, all images were visually checked for visible motion, and participants with motion were removed from the analyses. During preprocessing, we applied head motion correction with MCFLIRT (39), interleaved slice time correction, brain extraction of the functional image with BET (40), spatial smoothing with a Gaussian kernel of 5 mm full width. A high pass filter of 90 was applied. The functional images were coregistered to the participant's structural images that had been brain extracted with FSL's anatomical tool, using FMRIBs Linear Image Registration Tool (FLIRT) (39, 41), and to standard space (MNI152) using FMRIBs Non-Linear Image Registration Tool (FNIRT) (42), with a 10-mm warp resolution. Next, we applied independent component analysis (ICA)-based automatic removal of motion artifacts (ICA-AROMA) with the aggressive option to remove motion artifacts from the data through an ordinary least-squares regression (43, 44). In addition, we applied a custom-made script to remove white matter and cerebrospinal fluid from the signal and to improve registration. Registration of the functional to the structural images were visually inspected. Mean framewise displacements were calculated on the cleaned data using FSL's motion outlier command and used as covariates in the analyses. After all cleaning procedures, none of the participants had a mean framewise displacement higher than 0.03 mm.

#### Independent component analysis

**Figure 1.**
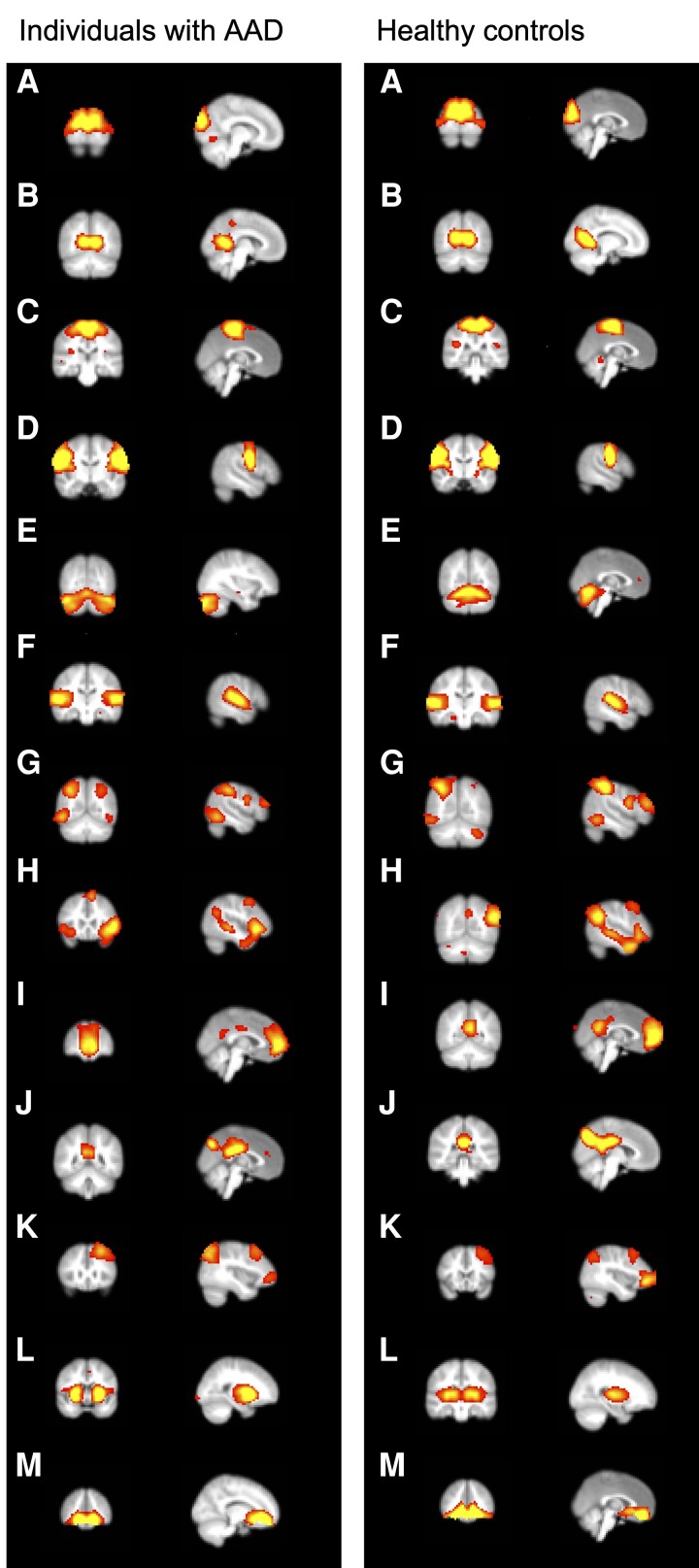
Identified resting-state networks in individuals with autoimmune Addison disease (AAD) (left) and healthy controls (right). A, Visual pole; B, visual medial; C, sensorimotor (motor leg-hand); D, motor face regions; E, cerebellum; F, auditory/language; G, dorsal attention; H, salience; I, default mode network (anterior); J, default mode network (posterior); K, frontoparietal/central executive (CEN); L, basal-ganglia (subcortical); M, orbitofrontal cortex.

rs networks were extracted using spatial independent component analysis on the cleaned data of each participant using MELODIC v 3.15 software (FSL) (45). For visualization purposes we first ran the MELODIC ICA for the groups separately and present the networks identified in the patient group and controls in Fig. 1. Next, we concatenated the data of all participants and ran the MELODIC ICA on all individuals in our cohort to identify the networks common to the whole cohort. These networks were fed into the dual regression analyses. The number of components was set to 60 networks. All spatial maps of the networks, time courses, and power spectra were visually inspected by experienced neuroimaging researchers (A.W. and N.P.) to classify them based on spatial similarity to the functional networks described in healthy people (46).

#### Dual regression analysis

##### Main group comparison

The obtained ICA components were regressed back into individual space using dual regression (45). Group comparisons were then performed using the FSL's randomize tool (47) with 5000 permutations, to identify differences in rs-fc between patients with Addison disease and controls. Sex, age, and mean framewise displacement were used as covariates. We also ran interaction analyses between the diagnostic group and sex on all clusters. Significant clusters were identified with threshold-free cluster enhancement with a significance threshold of *P* less than .05 and an arbitrary minimum cluster size of 40 voxels (47). We did not conduct an a priori power calculation, but our sample far exceeds the recommend size of at least 24 participants per group for fMRI analyses to detect signal changes of 0.5% with a power of 80% (48, 49).

##### Disease-related factors

We were interested in the relationship between disease related factors, namely GC replacement dose in mg HC eq/m^2^/day, number of experienced adrenal crises, age at diagnosis, and disease duration in years, and rs-fc in the networks where we initially observed a main group difference. We therefore assessed the relationship between these variables and rs-fc in 3 networks that were found to be significantly different between patients and controls in the main dual regression analyses, namely the orbitofrontal cortex (OFC), posterior DMN, and medial visual network, within the patient group separately, correcting for sex, age, and mean framewise displacement.

##### Associations with mental fatigue and executive function

First, we used linear regression to test the difference in mean score on the tests of interest, as well as the interaction between group and sex. For the MFI scales we added sex and age at testing as covariates. We tested if the association between rs-fc in any of the networks and mental fatigue, self-reported executive function problems, or performance on working memory tasks differed between patients and controls. We decided to assess all networks for 3 reasons: 1) to avoid circular testing, 2) because the relationship between rs-fc and functional outcome might differ between patients and controls without a main group difference in rs-fc of those regions per se, and 3) we did not have a specific a priori hypothesis about which networks would be differentially associated with fatigue and executive functions. We performed interactions between the diagnostic group and the variables of interest with rs-fc of all networks as an outcome, while correcting for sex, age, and mean framewise displacement. For the networks in which a significant interaction was found, we performed post hoc tests to assess the relationship between the relevant variable and rs-fc in that network in the patient and control groups separately.

## Results

### Cohort

Patients with AAD were on average 3 years older compared to control participants (*P* = .031), but there was no difference between patients and controls in the distribution of sex, education level, or alcohol use (Table 1). There were no sex differences in the patient group for dose of GC or MC medication, use of IR-HC or MR-HC, disease duration, age at diagnosis, or number of adrenal crises (Table 2).

**Table 1.** Background data of all participants

Variable		AAD (n = 57)	Control (n = 69)	*P*
Sex	% Female	58%	56%	≥.999
Age	Mean (SD) Range	32.6 (6.6) 19-42	29.6 (7.5) 19-43	.031
Education*^a^*	% Higher educated	46%	36%	.698
Alcohol*^b^*	No. per week	1.3	1.3	.672

Wilcoxon test for nonparametric data is applied for group differences in age and alcohol use. Chi-square test is applied for group differences in sex and education level.

Abbreviation: AAD, autoimmune Addison disease.

^*a*^Percentage of participants who have completed at least 3 years of university education.

^*b*^Number of times per week the individual used to consume alcohol.

**Table 2.** Medication use and disease characteristics of the patients with autoimmune Addison disease

Type of GC	All, n = 57	Female, n = 33	Male, n = 24	*P* (female vs male)
IR-HC (n)	45	25	20	–
GC dose (mg/m^2^/d)*^a^* (mean (SD))	13.3 (3.5)	13.2 (3.5)	13.4 (3.6)	.825
Doses/d (mean (SD))	2.6 (0.7)	2.6 (0.7)	2.6 (0.7)	.849
MR-HC (n)	12	8	4	–
GC dose (mg/m^2^/d)*^b^* (mean (SD))	12.5 (2.7)	11.5 (2.3)	14.5 (2.4)	.082
Doses/d (mean (SD))	1.3 (0.7)*^c^*	1.3 (0.5)	1.5 (1.0)	.662
Florinef, median dose in mg (range)	0.1 (0.05-0.25)	0.1 (0.05-0.25)	0.1 (0.05-0.20)	.411
**Disease characteristics**				
Age at AAD diagnosis, y (mean (SD)) Median Range	22.9 (6.7) 23 8-34	23.3 (6.6) 23 10-34	22.5 (7.1) 21.5 8-34	.650 – –
AAD duration, y (mean (SD)) Median Range	9.6 (4.9) 9.8 2.5-24.9	9.6 (4.0) 9.9 2.5-18	9.6 (6.0) 8.5 2.8-24.9	.999 – –
No. of adrenal crises (mean (SD)) Median Range	3.2 (5.1) 1 0-25	3.9 (5.6) 1 0-25	2.3 (4.2) 1 0-20	.221 – –
Hypothyroidism	43.9%	48.5%	37.5%	.579

*P* values indicate comparisons between male and female patients; *t* tests were used except for the occurrence of hypothyroidism, for which a chi-square test was used. From those on IR-HC replacement therapy, 2 patients had only 1 dose per day, 18 had 2 doses per day, 22 had 3 doses per day, and 3 patients had 4 doses per day.

Abbreviations: AAD, autoimmune Addison disease; GC, glucocorticoid; IR-HC, immediate-release hydrocortisone; MR-HC, modified-release hydrocortisone.

^*a*^Total IR-HC dose per day in mg per m^2^ of body surface (total mg/sqrt (cm × kg/3600)).

^*b*^Total IR-HC equivalence dose per day in mg per m^2^ of body surface (total mg/sqrt (cm × kg/3600)).

^*c*^Some participants on MR-HC take additional IR-HC doses on demand.

### Independent Component Analyses

From the 60 networks initially included, 13 were deemed to be real rs networks, while the others were discarded. The 13 rs networks identified in the patient and control groups separately are shown in Fig. 1.

### Dual Regression Analyses

#### Main group comparisons

**Figure 2.**
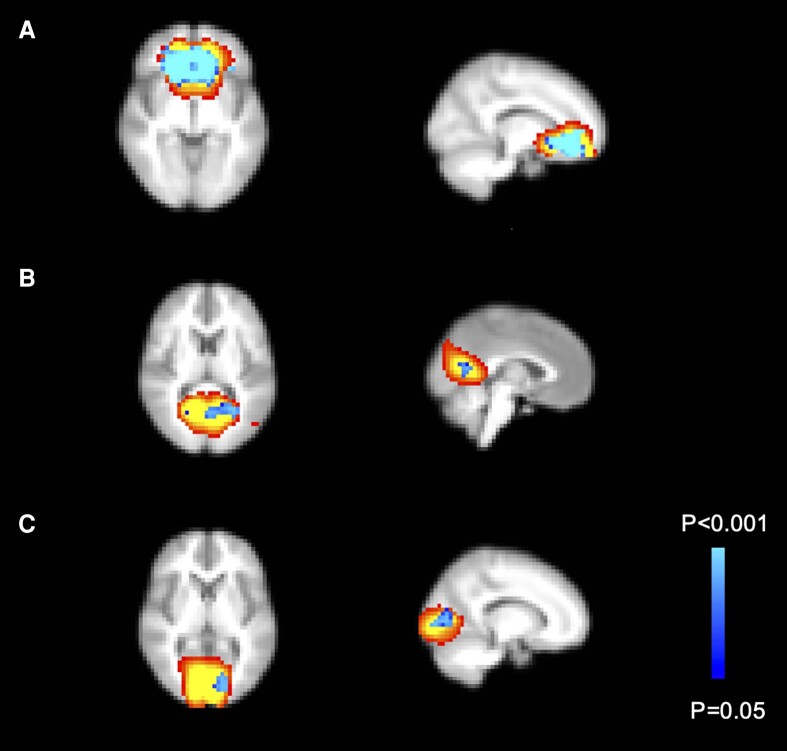
Networks with significantly increased resting-state functional connectivity (rs-fc) in individuals with autoimmune Addison disease (AAD) compared to controls. Individuals with AAD had increased rs-fc in the A, bilateral orbitofrontal cortex; B, left precuneus and lingual gyrus; and C, left intracalcarine cortex and lingual gyrus.

There was no significant group difference in framewise displacements (β = .002; *P* = .082). Participants with AAD had increased rs-fc in 3 main clusters belonging to 3 networks: the bilateral medial OFC (orbitofrontal network), the left precuneus and lingual gyrus (posterior DMN), and left intracalcarine cortex/part of the lingual cortex (medial visual network) (Fig. 2 and Table 3). There were no interactions between diagnostic group and sex for any of the networks. These findings remained when subanalyses were performed excluding patients on selective serotonin reuptake inhibitor treatment (n = 7), correcting for IQ, or correcting for time of the day when scanned.

**Table 3.** Cluster information for all significant findings

Region	No. of voxels	Coordinates cluster peak
**Main comparison between patients with Addison disease and controls** Patients > Controls		
Bilateral orbitofrontal cortex	775	X = −22, Y = 26, Z = −20
Left precuneus/lingual gyrus	88	X = 6, Y = −70, Z = 4
Left intracalcarine cortex/lingual gyrus	51	X = −14, Y = −90, Z = 0
**Interaction between diagnostic group and mental fatigue**		
Left inferior lateral OCC Right lingual gyrus	74 68	X = −42, Y = −82, Z = −12 X = 14, Y = −82, Z = −8
Left intracalcarine cortex	66	X = −14, Y = −78, Z = 12
**Interaction between diagnostic group and general fatigue**		
Left intracalcarine cortex	79	X = −2, Y = −82, Z = 16
Left inferior lateral OCC	58	X = −42, Y = −78, Z = 12
**GC dose associated with rs-fc within patient group** Positive associations:		
Left orbitofrontal cortex	42	X = −30, Y = 38, Z = −4

Abbreviations: GC, glucocorticoid; OCC, occipital cortex; rs-fc, resting-state functional connectivity.

#### Within-group associations between disease-related factors and resting-state functional connectivity

**Figure 3.**
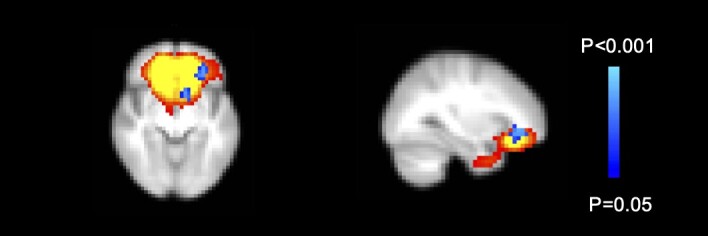
Glucocorticoid medication dose associated with resting-state functional connectivity (rs-fc) in patients with autoimmune Addison disease. A higher glucocorticoid dose in mg hydrocortisone eq/m^2^/day was associated with stronger rs-fc in a small part of the left orbitofrontal cortex (42 voxels).

GC replacement dose (in mg HC eq/m^2^/day) was associated with stronger rs-fc in only a small part of the left OFC (Fig. 3).

Age at time of diagnosis, disease duration, number of patient-reported adrenal crises, and minutes since last intake of GC medication were not associated with rs-fc in the 3 networks in which a main group difference was observed.

#### Interactions between diagnostic group and mental fatigue and executive functions for resting-state functional connectivity in all networks

In the cohort of this manuscript, which is smaller than the original cohort on which we published our cognition results (19), individuals with Addison scored lower on the working memory task forward (*P* = .041), reported more general fatigue (*P* = .017), and there was an interaction between sex and diagnosis for mental fatigue, with only female participants with Addison disease reporting more mental fatigue compared to female controls (*P* = .007) (Table 4).

**Table 4.** Executive functions and mental fatigue scores for patients and controls and split by sex

Whole group	AAD (n = 57) Mean (SD)	Control (n = 69) Mean (SD)	*P* AAD vs control	*P* Diagnosis × Sex
WAIS-IV, Digit Span (S)	10.21 (2.02)	10.97 (2.36)	.058	.132
WMS-III, Span Board (Forward) (S)	10.02 (2.87)	11.04 (2.67)	.041*^a^*	.264
WMS-III, Span Board (Backward) (S)	11.32 (2.31)	11.97 (1.72)	.072	.462
BDEFS-SF total	32.11 (8.69)	30.51 (7.80)	.283	.107
MFI Mental Fatigue	11.25 (3.67)	10.20 (2.99)	.169	.008*^b^*
MFI General Fatigue	12.67 (3.87)	10.54 (4.11)	.017*^a^*	.783

Split by sex	Female		Male	
	AAD (n = 33) Mean (SD)	Control (n = 39) Mean (SD)	AAD (n = 24) Mean (SD)	Control (n = 30) Mean (SD)
WAIS-IV, Digit Span (S)	10.58 (2.15)	10.82 (1.96)	9.71 (1.76)	11.17 (2.84)
WMS-III, Span Board (Forward) (S)	10.27 (3.04)	10.82 (2.93)	9.67 (2.63)	11.34 (2.29)
WMS-III, Span Board (Backward) (S)	11.70 (1.98)	12.13 (1.47)	10.79 (2.65)	11.76 (2.01)
BDEFS-SF total	34.48 (9.50)	30.90 (6.43)	28.83 (2.65)	30.00 (9.43)
MFI Mental Fatigue	12.45 (3.47)*^a^*	9.87 (2.70)*^a^*	9.58 (3.31)	10.61 (3.35)
MFI General Fatigue	13.48 (3.82)	11.22 (4.12)	11.54 (3.72)	9.67 (4.04)

Abbreviations: AAD, autoimmune Addison disease; BDEFS-SF, Barkley Deficit in Executive Functioning Scale—Short Form; MFI, Multidimensional Fatigue Inventory; S, scaled score, population norm M = 10, SD = 3; WAIS, Wechsler Adult Intelligence Scale; WMS, Wechsler Memory Scale.

^*a*^ *P* less than .05.

^*b*^ *P* less than .01.

There was an interaction between diagnostic group (AAD patients; healthy controls) and mental fatigue for rs-fc within the left inferior lateral occipital cortex (OCC) (lateral visual network), right lingual gyrus (lateral visual network), and left intracalcarine cortex (medial visual network). General fatigue was also differentially associated with rs-fc within the left lateral inferior OCC (lateral visual network) and the left intracalcarine cortex (medial visual network) between patients and controls (see Table 3). However, post hoc analyses did not reveal significant associations between mental nor general fatigue, and rs-fc in these regions in patients or controls separately. When investigating females only, there were no interactions between diagnostic group and mental fatigue in any of the networks.

There were no interactions between diagnostic group and visuospatial working memory, and self-reported executive functioning (BDEFS total, self-organization and emotion regulation) in any of the networks.

## Discussion

In this first study assessing rs-fc in individuals with AAD, patients had increased rs-fc within 3 major networks, namely the OFC, the posterior DMN (precuneus), and the medial visual network (intracalcarine cortex and lingual gyrus). Being on a relatively higher GC replacement dose was associated with stronger rs-fc in a small part of the OFC network.

The observations confirm our hypothesis that having AAD affects the baseline functional organization of the brain. Whether the alterations in rs-fc are a compensation mechanism or a maladaptive result of the mild whole-brain atrophy that we found in this cohort, or an independent effect, remains to be investigated (21). The difference in rs-fc partly corresponds to the regions that tended to show the strongest reduction in volume in patients with AAD, in particular the OFC (21). Although anatomic architecture provides the condition for function, alterations in function are not necessarily determined solely by the anatomic architecture and can have independent effects on cognition (50, 51).

As this is the first assessment of brain activity in AAD, we have no previous studies to compare our findings with. However, in our investigation of patients with CAH we also observed stronger rs-fc in patients compared to healthy controls in the precuneus (29). Other groups have found increases in rs-fc in patients with Cushing disease in several networks partially overlapping the ones we identified here, including the posterior cingulate cortex/precuneus and the prefrontal cortex (26), the subgenual ACC-DMN (27), and the medial temporal (right parahippocampal gyrus) and medial prefrontal cortex (specifically the ACC and bilateral orbital gyrus (28)). These primary visual and posterior and anterior DMN networks thus seem to be particularly sensitive to disturbances in cortisol regulation. Although increased rs-fc might specifically be a result of excess cortisol levels, results from other groups investigating Cushing patients also suggest that this may be region specific (28). Within our AAD patient group, a relatively higher GC replacement dose was associated with stronger rs-fc in part of the network in the OFC that differed between patients and controls. Previously we found that a higher GC dose was also associated with somewhat smaller total BV (21). How these findings are exactly related to each other is not yet clear. It is important to further investigate this question, as our findings indicate that GC dosing may affect brain health in the long run.

The networks in which we found a significant group difference—the medial visual, DMN, and OFC—are involved in a great variety of tasks. The intracalcarine cortex and lingual gyrus are part of a primary visual network that processes visual information (52), while the DMN is a major rs network and has been associated with, among others, self-referential thinking (53, 54). The precuneus in particular is one of the major hubs of this network and has a high metabolic demand (55). Reduced DMN suppression is associated with rumination in patients with major depression, including both parietal and ventromedial prefrontal parts of the DMN (56). Of the 3 networks we found, the OFC is the most known for containing a high density of GC receptors (57). The OFC, or ventromedial prefrontal cortex, is involved in many higher-order cognitive processes such as motivation and emotion regulation (58), processing reward-related information needed for emotional and social behavior (59), but also shaping autonomic and endocrine responses (60). Aberrant activity in this area is associated with depression and sleep problems (61). In the latter study, changes in functional connectivity in the ventromedial prefrontal cortex mediated the association between depressive problems and poor sleep quality (61). This could mean that stronger rs-fc in the OFC in AAD could predispose to emotion-regulation problems, potentially as a result of mental fatigue or sleep disturbances. This has been found in Cushing diseases where altered activity in this area was found during emotion processing (62). However, we did not find any associations between rs-fc in this region and self-reported emotion-regulation problems (a subscale of the executive function self-report), nor with mental fatigue or self-reported sleep quality. Of note, it is also possible that patients were more worried during MR scanning and were therefore ruminating more and as a result had stronger connectivity in DMN hubs.

Our hypothesis that changes in rs connectivity might be related to mental fatigue or the experience of executive function problems was not confirmed. Although both general and mental fatigue were differentially associated with rs-fc in patients compared to controls in several visual networks, including the calcarine cortex and lingual gyrus, we did not find any meaningful relationships between fatigue and rs-fc in those areas when looking at the groups separately. This is rather surprising, as mental fatigue has been associated with increases in task and rs activity and rs connectivity in particular in the DMN and visual areas (30, 63–66). These studies suggest that fatigue causes a shift in energy expenditure toward baseline self-referential processing as opposed to task-relevant networks (65). Increased rs-fc has also been found at baseline as a compensation mechanism for gray and white matter atrophy that may protect people from getting fatigued, as was found in multiple sclerosis patients (67). However, as we found no correlation between rs-fc and fatigue in any direction within the patient group, we cannot conclude that the increase in rs-fc was part of a compensation mechanism for patients. Potentially the difference in fatigue was not strong enough to find significant associations with rs network connectivity. Moreover, there was a significant interaction with sex, whereby only female patients reported more mental fatigue than controls. A larger study is needed to investigate the relationship between fatigue and brain activity specifically in females.

We found no group differences in the relationship between rs-fc and any of the cognitive functions. Thus, rs-fc did not explain the self-reported problems with executive functioning (19). Possibly, increased rs-fc is part of a compensation mechanism (27). Such changes in the functional organization of the brain as a response to changes in structure to maintain cognitive ability is part of the brain’s metastability (68). However, lack of variation in cognitive performance and rs-fc within our patient group might have obscured associations between rs-fc and executive functioning that could support a hypothesis for either a compensation mechanism or a maladaptive response. The group difference in working memory performance was small and significant for only one task. We did not replicate our previous finding of more self-reported executive functioning difficulties, which is likely due to reduced power in this smaller sample with good-quality rs images. Hence, in this sample the relative lack of performance and self-report difference may explain the lack of association with rs outcomes. Longitudinal studies designed to differentiate high-performing from less-well performing patients are needed to further understand our findings, to follow the brain development over time, and to establish whether the observed alterations are contributing to metastability or lead to problems later on.

The mechanism underlying the observed increases in rs-fc in patients is unknown. Some clues are found in studies of other diseases. For example, in Cushing patients, shorter time since remission and blood cortisol levels were associated with strength of rs-fc in the patients, suggesting that higher cortisol levels are related to increased rs-fc (26–28). Moreover, in Cushing disease patients there was a correlation between cortisol and brain glucose metabolism (69), which corresponds to the idea that connectivity in the DMN is associated with metabolic activity (55). Indeed, there seems to be a (nonlinear) relationship between brain glucose consumption and functional connectivity, for example, in the visual cortex (70). Thus, the steep peaks in cortisol when patients take their medication could potentially lead to increased glucose consumption in hubs of the DMN and visual cortex. The steep morning rise in cortisol might specifically increase rs-fc, as was found in the medial frontal cortex of healthy people in the afternoon (71). Cortisol itself may also affect neuronal excitability directly, though not necessarily on a neuronal population level (72). The relationship between cortisol-related metabolic changes and direct effects on neuronal excitability and changes in rs connectivity signal are therefore not yet clear. Despite this, our results show that individuals with AAD do have changes in rs-fc in various networks, and follow-up studies are needed to evaluate how this develops over time and if changes in rs-fc over time are related to changes in cognitive functioning, experienced executive functioning problems, and mental and general fatigue. To maintain brain health, a GC replacement therapy that better mimics natural cortisol secretion would probably be beneficial. Indeed, we propose that both psychological and brain health need to be considered when optimizing replacement medication.

### Limitations

Although this study benefits from a relatively large sample, some limitations need to be noted. First, we did not fix the time during the day when participants were scanned, nor the time since the patients took their last medication dose. Hence, we may have introduced variability that could have diluted the effects. In addition, the patient group was 3 years older on average. However, we included age as a covariate in the analyses, so this difference may not have affected the results to a large extent.

## Conclusion

Individuals with AAD have stronger rs-fc in several brain networks, partly correlating with GC replacement dose. Future studies are needed to determine if these changes predispose individuals to problems with cognition and mood later in life or if they are part of a compensation mechanism.

## Data Availability

The data sets generated during and/or analyzed during the current study are not publicly available but are available from the corresponding author on reasonable request.

## Abbreviations

AAD: autoimmune Addison disease
ACC: anterior cingulate cortex
BDEFS-SF: Barkley Deficit in Executive Functioning Scale—Short Form
BV: brain volume
CAH: congenital adrenal hyperplasia
DMN: default mode network
fMRI: functional magnetic resonance imaging
GC: glucocorticoid
ICA: independent component analysis
IR-HC: immediate-release hydrocortisone
MC: mineralocorticoid
MFI: Multidimensional Fatigue Inventory
MR-HC: modified-release hydrocortisone
MRI: magnetic resonance imaging
OFC: orbitofrontal cortex
rs-fc: resting-state functional connectivity
